# Preliminary design for establishing compost maturity by using the spectral characteristics of five organic fertilizers

**DOI:** 10.1038/s41598-022-19714-3

**Published:** 2022-09-20

**Authors:** Yi-Hong Lin, Yong-Zhang Lin, Yong-Hong Lin

**Affiliations:** 1 Department of Mechanical Engineer, National Pingtung University of Science and Technology, Pingtung, Taiwan; 2 Department of Plant Industry, National Pingtung University of Science and Technology, Pingtung, Taiwan

**Keywords:** Biotechnology, Environmental sciences

## Abstract

The maturity of compost is involved in the availability of nutrients to crops and improvement of soil properties after fertilization. In the past, the determination of composts maturity mostly required analysis in the laboratory previously and it must consume a lot of time and cost. This study was conducted to use Fourier Transform Infrared (FTIR) spectroscopy and solid ^13^C Nuclear Magnetic Resonance (^13^C NMR) spectroscopy to understand the mature characteristics of five type of common composts. The FTIR analysis showed that all composts contained aromatic groups. In addition, the surface of five composts contained the functional groups including hydroxyl group, carboxyl group, amino group etc. However, these functional groups changed along with maturity degree. It is recognized that the aliphatic group located at 2930 cm^−1^ and 2850 cm^−1^ showed a decreasing peak, and amino acid at 1385 cm^−1^ was disappearing gradually due to the decomposition of organic matter by bacteria. There may be used to identify the maturity degree of composts. Increase of aromatic group at 1650 cm^−1^, carboxy (–COOH) and phenolic OH group at 1385 cm^−1^ may prove the full maturity of composts. ^13^C NMR analysis showed that five type of matured composts are mainly consisted with aliphatic groups and aromatic groups. The surfaces of the composts contained C–O bonds (ester, ethers, carbohydrate and other functional groups), COO^−^ (carboxyl and ester carbons) and C=O bond (aldehydes and ketones). The strength of different absorptive characteristics of FTIR and ^13^C NMR may be a clue to identify the maturity of composts for the design of detective instruments in the future.

## Introduction

Organic agriculture has been paid more attention and the application of organic fertilizer was gradually increased recently^1^. Organic fertilizer is produced through composting of complex organic materials which are decomposed by microorganism and converted to simple and stable components as fertilizer^2,3^. Mature compost can serve nutrients quickly for plants after application due to its low C/N ratio^4^. Application of mature compost can provide large amount of elements (N, P, K etc.) and trace elements (Fe, Mn, B etc.) to crops, and even inhibit activities of pathogen in the soil^5^. Humic acid is the important component of organic fertilizer. Carboxylic and phenolic groups in humic acid containing OH bonds which are the sites for bonding with metals^6,7^.

Traditional index for evaluation of compost maturity includes pH (Joyce et al., 2010)^8^, electrical conductivity (EC)^4^, C/N ratio^5,8^, humic acid (HA) and fulvic acid (FA) ratio^3,5^ and germination rate of vegetable seeds. Generally, several indices are combined to judge the maturity of compost^9^. However, these methods mostly require chemical and biological analysis in the laboratory, and these procedures must consume a lot of time and cost, and the disposal of chemicals will cause pollution of environment.

In the past there were many successful examples of studies using FTIR and ^13^C NMR analysis^8^. These methods are also widely used in medicine, foods and engineering, etc. In agriculture they are also used for the examination of timber quality^10^, germination rate of rice^11–13^ and humic acid characteristics^14^. These methods are highly correlated positivity with traditional chemical or biological methods of analysis^5^. They are not only saving time and labor but also reducing use of chemicals that pollute environment in comparison with the traditional chemical analytical methods. The application of composts will be beneficial for the improvement of soil properties and the supply of available nutrition for plants^15–17^. However, the maturity of compost is crucial for these benefits^18,19^. Chen et al. (1989) had ever showed that the measurement of composts by ^13^C NMR and FTIR on the decomposition of organic matter will provide full message on the characteristics of composts. However, their experimental material was only the cattle manure. Rare researches were focused on the characteristics of different composts during maturing procedure by the analysis of spectrum. In this research, Fourier Transform Infrared (FTIR) spectroscopy and ^13^C Nuclear Magnetic Resonance (^13^C NMR) were used for understanding the variation of functional groups on the surface of five commonly applied composts. It maybe serve the reference to design spectral instrument for detecting the maturity of different composts in the future.

## Materials and methods

### Producing and sampling of composts

Five composts were produced using commonly available materials were chicken manure, pig manure, cattle manure, lemon peel waste and soybean meal. Every kind of material was mixed with sawdust at the ratio of 3–1, respectively. The moisture were adjusted to 55–60% and the thermometer were inserted into the middle layer of composts. They were turned over twice a week. Beginning at third week, the compost was turned over once a week. The moisture of composts were monitored with tensiometer and controlled at 60–65%. The properties of five composts were sampled and determined at 1, 2, 4, 6 and 8 week after composting, respectively. The FTIR analysis was proceeded at 0, 3, 6 and 9 week after composting, respectively. The ^13^C NMR analysis of five composts were proceeded for the finished products.

### Measurement of composts

Carbon (C) of compost was measured using element analyzer (Elementar vario EL III). N was measured following Kjeldahal method^20^. The C/N ratio was then calculated. pH of compost was determined by grass electrodes after saturating with water and stirred thoroughly 21^16^. 100 g oven-dried sample was ashed in an oven at 600 °C for 24 h, the weight loss of compost was organic matter content and expressed in percentage. 0.2 g oven-dried sample was digested by H_2_SO_4_ and then measured K (potassium), Ca (calcium), Mg (magnesium), Fe (iron), Mn (manganese), Cu (copper) and Zn (zinc)^21,22^ using inductively coupled plasma spectrometer (ICP, JY Ultima2). Phosphorus was extracted by Bray No. 1 method and measured following Molybdenum method^23^.

### Measurement of humic acid

After oven-drying at 105 °C for 24 h, the 10 g of organic fertilizer was weighed into the flask and added 100 ml of 0.1 N NaOH^23^, and then it was shaken for 24 h. The supernatant solution was centrifuged and collected for the precipitated material. The extraction procedure was repeated three times by 50 ml of extractant every time for extracting complete humic substances in the sample. The humic substances were treated by HCl-HF mixture for 24 h and then was centrifuged as described by Stevenson (1981)^24^. The residue was freeze-dried for acquiring pure humic acid.

### FTIR measurement on compost

FTIR analysis was based on the method of Silverstein et al. (1981). 1 mg sample was grounded by an agate motor and added into 200 mg KBr that was pre-dried at 110 °C and mixed well before put in a die. The die was then rotated several times with 10,000–15,000 lb/m^2^. After above mention, it was preserved for 3 min under air-pressurization. The sample was now contained in KBr pellet. Fourier Transform Infrared spectrophotometer (FTIR) (Shimazu, Japan) was used to measure light transmissivity at wave number from 4000 to 400 cm^−1^^25^.

### ^13^C nuclear magnetic resonance spectrophometer analysis

One gram of sample was put in a grass tube of 4 cm long with 10 mm inner diameter and subjected to solid Nuclear Magnetic Resonance spectrophotometer (MSL-200 NMR type, Germany) measurement under super conductive magnetic field. The spectral frequence of the instrument was 50.33 MHz with 1 ms retardation time (acquisition time, delay time) and 1 s recycle time. The magic angle spinning rate was about 3.5 kHz. The spectra were separated into 0–50, 50–90, 90–110, 110–140, 140–160 and 160–190 ppm sections based on chemical shift in order to show single intensity of different types of carbon. Relative content of each carbon chemical structure was integrated by a computer program. The area of each section was calculated and expressed in its percentage^26,27^.

## Results

### The characteristics of five composts

During composting procedure, chicken manure showed its temperature raise to 65–75 °C after 3 days. The compost was turned over twice times in the first week and changed to one time from the second week. Compost samples were collected at 0, 2nd, 4th, 6th and 8th week for analysis. Table 1 showed that pH of chicken manurewas 7.8 at first. However, it decreased along with composting time and reached about 7.1 after 8 weeks. The organic matter content was 92.5% originally and decreased to 64.0% after 8th week composting, however, the humic acid increased following composting time 31^24^. Carbon content of chicken manure was decreased from 58.1 to 43.3% and nitrogen content decreased from 2.45 to 2.2%. By way of calculating, C/N ratio decreased from 23.7 to 19.7, indicating it was mature gradually. Commonly, the compost was mature in case of C/N ratio below^20,28^. On the other hand, Increasing with composting time, P, K, Ca, Mg, Fe, Mn, Cu, Zn and Na all gradually increasing. Table 2 showed pH of cattle manure was 7.3 at first and reduced to 7.1 after 8 week composting. Organic matter content decreased from 91.3 to 55.2% and humic acid increased from 1.91 to 3.25% after 8 weeks composting. C/N ratio decreased to below 20, representing that the cattle manure was matured. P, K, Ca, Mg, Fe, Mn, Cu, Zn and Na contents of cattle manure were all significantly increased following composting time. Table 3 showed that pH of pig manure was 7.2 before composting and decreased from 88.8 to 61.1% and humic acid increased from 0.7 to 2.5%. Carbon content decreased from 61.2 to 48.6% and nitrogen increased from 23.3 to 2.45% after composting 8 week. Contents of P, K, Ca, Mg, Fe, Mn, Cu, Zn and Na all increased significantly following composting time. C/N ratio decreased from 26.3 to 19.8. After 8 weeks, the ratio was below 20 which representing the pig manure was mature. Table 4 showed that pH was slighly acidic for soybean manure before composting, however, it decreased following composting time. The organic matter content decreased from 90.2 to 67.0% after 8 weeks. Humic acid increased from 0.47 to 1.26%. The content of carbon decreased from 55.1 to 47.0% and nitrogen increased from 5.04 to 5.33%. The contents of P, K and Na were not sable along with composting time. There was an increasing trend for Ca, Mg, Fe, Mn, Cu and Zn. As soybean manure contained high nitrogen content, the C/N ratio was below 20 originally and decreased slightly along with composting time. Table 5 showed that pH of lemon peel waste was slightly acid before composting and had an increasing trend was observed due to the decomposition of organic acid in the lemon peel. The organic matter decreased from 90.2 to 58.9% and humic acid increased from 1.3 to 4.9%. The content of carbon decreased from 71.7 to 58.1% and nitrogen increased slightly from 2.62 to 2.94%. There was an increasing trend for P, K, Ca, Mg, Fe, Mn, Cu, Zn and Na following composting time. The C/N ratio was decreased from 27.4 to 19.8 after 8 weeks, hence, the lemon peel compost was mature.

**Table 1:** The chemical properties of chicken manure at different sampling stage.

Sampling time	pH	OM^3^ (%)	HA^4^ (%)	C/N	C (%)	N (%)	P (%)	K (%)	Ca (%)	Mg (%)	Mn (%)	Fe (%)	Cu (%)	Zn (%)	Na (%)
W0^2^	7.8 ± 0.2^a1^	92.5 ± 5.3^a^	1.8 ± 0.3^b^	23.7 ± 1.5^a^	58.1 ± 3.1^a^	2.45 ± 0.3^a^	0.89 ± 0.21^b^	3.08 ± 0.43^b^	4.15 ± 0.49^ab^	0.83 ± 0.21^c^	478 ± 89^b^	607 ± 183^c^	78 ± 32^b^	597 ± 137^b^	5257 ± 1395^a^
W2	7.6 ± 0.3^a^	88.6 ± 2.5^a^	2.3 ± 1.5^ab^	23.0 ± 1.3^a^	55.4 ± 2.8^a^	2.40 ± 0.5^a^	0.78 ± 0.18^b^	2.82 ± 0.48^b^	3.26 ± 0.28^b^	0.90 ± 0.19^c^	387 ± 107^b^	628 ± 231^c^	86 ± 21^b^	695 ± 184^b^	5174 ± 1284^a^
W4	7.6 ± 0.2^a^	79.3 ± 6.8^ab^	3.2 ± 0.8^ab^	21.6 ± 1.3^a^	50.5 ± 3.0^a^	2.34 ± 0.3^a^	1.42 ± 0.47^a^	4.12 ± 0.36^a^	4.14 ± 0.45^ab^	1.78 ± 0.18^b^	1008 ± 188^a^	2532 ± 296^b^	136 ± 29^a^	1318 ± 333^a^	7226 ± 492^a^
W6	7.3 ± 0.3^a^	68.8 ± 1.2^b^	4.1 ± 0.7^a^	20.6 ± 0.2^a^	47.6 ± 1.3^ab^	2.31 ± 0.4^a^	1.47 ± 0.22^a^	4.18 ± 0.45^a^	4.83 ± 0.58^a^	2.29 ± 0.30^a^	1197 ± 84^a^	4266 ± 288^a^	167 ± 33^a^	1550 ± 291^a^	7698 ± 587^a^
W8	7.1 ± 0.5^a^	64.0 ± 3.7^b^	4.9 ± 0.9^a^	19.7 ± 0.4^b^	43.3 ± 0.9^b^	2.20 ± 0.4^a^	1.87 ± 0.23^a^	4.25 ± 0.43^a^	5.64 ± 0.69^a^	2.94 ± 0.41^a^	1565 ± 275^a^	4565 ± 343^a^	129 ± 39^a^	1929 ± 235^a^	5856 ± 575^a^

^1^Different letters indicate significantly different results by LSD tests at *p* < 0.05.

^2^0, 2, 4, 6, 8 week after compost.

^3^Organic matter.

^4^Humic acid.

**Table 2:** The chemical properties of cattle manure at different sampling stage.

Sampling time	pH	OM^3^ (%)	HA^4^ (%)	C/N	C (%)	N (%)	P (%)	K (%)	Ca (%)	Mg (%)	Mn (mg/kg)	Fe (mg/kg)	Cu (mg/kg)	Zn (mg/kg)	Na (mg/kg)
W0^2^	7.3 ± 0.2^a1^	91.3 ± 10.2^a^	0.9 ± 0.2^c^	34.3 ± 3.9^a^	65.6 ± 4.6^a^	1.91 ± 0.18^b^	0.35 ± 0.18^b^	0.21 ± 0.06^b^	0.91 ± 0.43^b^	0.17 ± 0.17^b^	78 ± 42^c^	135 ± 57^c^	127 ± 39^c^	193 ± 46^d^	480 ± 33^c^
W2	7.2 ± 0.3^a^	88.2 ± 10.9^a^	1.3 ± 0.4^c^	25.8 ± 4.3^a^	62.2 ± 3.9^a^	2.41 ± 0.39^ab^	0.28 ± 0.20^b^	0.38 ± 0.05^b^	1.40 ± 0.38^b^	1.88 ± 0.15^b^	127 ± 14^c^	267 ± 125^bc^	215 ± 41^b^	323 ± 59^c^	898 ± 51^b^
W4	7.3 ± 0.2^a^	76.5 ± 6.5^ab^	2.5 ± 0.3^b^	23.4 ± 5.3^ab^	57.9 ± 4.1^a^	2.48 ± 0.38^a^	0.72 ± 0.17^a^	0.58 ± 0.10^a^	2.45 ± 0.29^ab^	1.97 ± 0.13^b^	216 ± 25^b^	485 ± 223^b^	264 ± 46^ab^	536 ± 125^b^	1209 ± 62^a^
W6	7.1 ± 0.3^a^	65.90 ± 5.3^b^	4.5 ± 0.4^a^	18.0 ± 2.7^b^	57.1 ± 4.8^a^	3.17 ± 0.35^a^	0.86 ± 0.22^a^	0.45 ± 0.10^a^	2.88 ± 0.28^a^	2.41 ± 0.28^a^	250 ± 33^ab^	2243 ± 188^a^	298 ± 43^ab^	639 ± 174^b^	906 ± 43^b^
W8	7.1 ± 0.3^a^	55.2 ± 5.1^b^	5.0 ± 0.7^a^	17.3 ± 3.2^b^	56.2 ± 4.6^a^	3.25 ± 0.34^a^	0.88 ± 0.24^a^	0.60 ± 0.13^a^	3.32 ± 0.11^a^	2.61 ± 0.25^a^	362 ± 38^a^	2683 ± 202^a^	367 ± 57^a^	1029 ± 201^a^	1134 ± 55^a^

^1^Different letters indicate significantly different results by LSD tests at *p* < 0.05.

^2^0, 2, 4, 6, 8 week after compost.

^3^Organic matter.

^4^Humic acid.

**Table 3:** The chemical properties of pig manure at different sampling stage.

Sampling time	pH	OM^3^ (%)	HA^4^ (%)	C/N	C (%)	N (%)	P (%)	K (%)	Ca (%)	Mg (%)	Mn (mg/kg)	Fe (mg/kg)	Cu (mg/kg)	Zn (mg/kg)	Na (mg/kg)
W0^2^	7.2 ± 0.2^a1^	88.8 ± 5.8^a^	0.7 ± 0.4^c^	26.3 ± 0.4	61.2 ± 1.6^a^	2.33 ± 0.12^a^	0.32 ± 0.07^b^	0.16 ± 0.03^b^	10,842 ± 1035^b^	1604 ± 166^c^	113 ± 61^b^	217 ± 96^d^	152 ± 49^c^	434 ± 123^c^	596 ± 93^b^
W2	7.1 ± 0.3^a^	85.9 ± 3.2^a^	1.4 ± 0.4^b^	26.0 ± 0.3^a^	58.1 ± 1.2^a^	2.23 ± 0.11^a^	0.47 ± 0.18^ab^	0.32 ± 0.04^a^	16,817 ± 988^b^	2751 ± 889^bc^	202 ± 32^b^	423 ± 175^c^	261 ± 56^b^	743 ± 139^ab^	1111 ± 166^a^
W4	6.8 ± 0.3^a^	78.1 ± 4.9^a^	1.6 ± 0.3^a^	24.1 ± 0.2^a^	54.9 ± 1.1^a^	2.28 ± 0.13^a^	0.68 ± 0.18^a^	0.35 ± 0.03^a^	25,773 ± 1125^ab^	3374 ± 955^b^	261 ± 18^b^	428 ± 236^c^	282 ± 58^b^	998 ± 83^b^	1427 ± 123^a^
W6	7.0 ± 0.1^a^	72.6 ± 5.8^b^	2.1 ± 0.3^a^	21.9 ± 0.2^ab^	51.9 ± 1.5^ab^	2.37 ± 0.12^a^	0.86 ± 0.16^a^	0.39 ± 0.04^a^	25,560 ± 1023^ab^	4914 ± 744^b^	308 ± 39^a^	1412 ± 1124^b^	278 ± 80^b^	1048 ± 90^b^	1203 ± 163^a^
W8	7.0 ± 0.2^a^	61.1 ± 5.4^b^	2.5 ± 0.5^a^	19.8 ± 0.4^b^	48.6 ± 2.4^b^	2.45 ± 0.14^a^	0.79 ± 0.15	0.32 ± 0.04^a^	38,424 ± 1321^a^	7361 ± 856^a^	406 ± 63^a^	4167 ± 1056^a^	568 ± 78^a^	2067 ± 89^a^	1057 ± 134^a^

^1^Different letters indicate significantly different results by LSD tests at *p* < 0.05.

^2^0, 2, 4, 6, 8 week after compost.

^3^Organic matter.

^4^Humic acid.

**Table 4:** The chemical properties of soybean manure at different sampling stage.

Sampling time	pH	OM^3^ (%)	HA^4^ (%)	C/N	C (%)	N (%)	P (%)	K (%)	Ca (%)	Mg (%)	Mn (mg/kg)	Fe (mg/kg)	Cu (mg/kg)	Zn (mg/kg)	Na (mg/kg)
W0^2^	6.8 ± 0.3^a1^	90.2 ± 15.2^a^	0.47 ± 0.13^b^	10.9 ± 0.9^a^	55.1 ± 2.3^a^	5.04 ± 0.15^a^	0.75 ± 0.25^b^	1.71 ± 0.11^a^	20,399 ± 3652^a^	6577 ± 2325^a^	111 ± 28^a^	258 ± 29^c^	17 ± 5^a^	96 ± 28^a^	3679 ± 126^a^
W2	6.6 ± 0.2^a^	86.8 ± 8.5^a^	0.62 ± 0.11^b^	9.8 ± 0.3^a^	54.8 ± 2.0^a^	5.14 ± 0.10^a^	0.72 ± 0.28^b^	1.72 ± 0.12^a^	16,324 ± 3254^a^	6332 ± 2946^a^	101 ± 29^a^	313 ± 39^c^	20 ± 3.1^a^	97 ± 22^a^	3513 ± 110^a^
W4	6.4 ± 0.2^a^	76.5 ± 6.1^ab^	1.11 ± 0.22^a^	9.8 ± 0.4^a^	54.1 ± 2.1^a^	5.18 ± 0.15^a^	1.33 ± 0.16^a^	1.64 ± 0.13^a^	15,876 ± 3891^a^	6960 ± 2896^a^	115 ± 12^a^	494 ± 101^bc^	18 ± 2.2^a^	106 ± 21^a^	3447 ± 113^a^
W6	6.2 ± 0.3^a^	75.1 ± 6.6^ab^	1.24 ± 0.18^a^	9.7 ± 0.4^a^	47.5 ± 3.2^a^	5.19 ± 0.15^a^	1.49 ± 0.19^a^	1.79 ± 0.15^a^	21,447 ± 2325^a^	9639 ± 1943^a^	154 ± 26^a^	716 ± 299^b^	19 ± 3.3^a^	136 ± 20^a^	3507 ± 102^a^
W8	6.2 ± 0.3^a^	67.0 ± 4.9^b^	1.26 ± 0.21^a^	9.1 ± 0.5^a^	47.0 ± 3.4^a^	5.33 ± 0.17^a^	1.21 ± 0.13^a^	1.60 ± 0.13^a^	22,625 ± 3310^a^	11,832 ± 3845^a^	167 ± 31^a^	1989 ± 142^a^	24 ± 3.5^a^	134 ± 18^a^	3128 ± 121^a^

^1^Different letters indicate significantly different results by LSD tests at *p* < 0.05.

^2^0, 2, 4, 6, 8 week after compost.

^3^Organic matter.

^4^Humic acid.

**Table 5:** The chemical properties of lemon peel compost at different sampling stage.

	pH	OM^3^ (%)	HA^4^ (%)	C/N	C (%)	N (%)	P (%)	K (%)	Ca (%)	Mg (%)	Mn (mg/kg)	Fe (mg/kg)	Cu (mg/kg)	Zn (mg/kg)	Na (mg/kg)
W0^2^	6.4 ± 0.3^a1^	90.2 ± 7.1^a^	1.3 ± 0.40^b^	27.4 ± .1.8^a^	71.7 ± 3.3^a^	2.62 ± 0.6^a^	0.23 ± 0.09^a^	1.98 ± 0.58^a^	16,029 ± 1011^c^	1022 ± 423^c^	9 ± 3.3^c^	177 ± 28^c^	5 ± 1.5^b^	11 ± 2.9^d^	380 ± 49^b^
W2	6.4 ± 0.2^a^	86.1 ± 5.3^a^	1.9 ± 0.8^b^	25.9 ± 1.1^a^	70.7 ± 2.8^a^	2.73 ± 0.5^a^	0.21 ± 0.98^a^	2.61 ± 0.43^a^	14,115 ± 1988^c^	2674 ± 233^b^	13 ± 2.7^c^	238 ± 44^b^	11 ± 3.4^a^	26 ± 3.4^c^	360 ± 43^b^
W4	6.3 ± 0.1^a^	76.5 ± 7.3^ab^	2.3 ± 1.0^ab^	27.1 ± 1.0^a^	62.6 ± 3.1^b^	2.29 ± 0.7^a^	0.32 ± 0.09^a^	2.07 ± 0.50^a^	18,657 ± 2315^c^	2288 ± 356^b^	22 ± 3.8^b^	301 ± 56^b^	12 ± 2.8^a^	35 ± 4.2^b^	301 ± 37^b^
W6	6.5 ± 0.2^a^	66.2 ± 3.8^b^	3.1 ± 1.1^b^	21.8 ± 1.4^b^	63.2 ± 2.8^b^	2.90 ± 0.6^a^	0.33 ± 0.10^a^	2.33 ± 0.47^a^	25,027 ± 3023^b^	4524 ± 610^a^	31 ± 7.1^b^	412 ± 71^b^	11 ± 3.1^a^	36 ± 5.1^b^	316 ± 42^b^
W8	6.7 ± 0.2^a^	58.9 ± 4.6^b^	4.9 ± 0.5^a^	19.8 ± 1.4^b^	58.1 ± 3.3^b^	2.94 ± 0.4^a^	0.32 ± 0.08^a^	2.33 ± 0.42^a^	51,256 ± 4610^a^	5746 ± 689^a^	63 ± 6.5^a^	988 ± 55^a^	16 ± 3.8^a^	63 ± 3.8^a^	534 ± 39^a^

^1^Different letters indicate significantly different results by LSD tests at *p* < 0.05.

^2^0, 2, 4, 6, 8 week after compost.

^3^Organic matter.

^4^Humic acid.

### FTIR analysis in five composts at different composting time

Through FTIR analysis, the kind and strength of functional groups from different sources of compost. Table 6 showed that the absorption degree (%) of wave number (cm^−1^) at the sampling stage of different manures. Following composting time, it was decreased at wave number 1450, 1600 and 3050 cm^−1^ for chicken manure. 1400, 1650 and 3000 cm^−1^ for cattle manure. 1380, 1580 and 2930 cm^−1^ for pig manure. 1350, 1480 and 2900 cm^−1^ for soybean manure. 1320, 1460 and 2950 cm^−1^ for lemon peel compost. Table 7 showed the types of functional groups and their strength as determined by FTIR analysis on different composts. The strongly functional groups were as follows. OH group, C=C bonding, C–O or –OH(1160 cm^−1^) in the chicken manure and cattle manure, OH group and C=C bonding in the pig manure, C=C bonding in the soybean manure and lemon peel compost.

**Table 6:** The absorption of FTIR spectroscopy of main wave number at the sampling stage of different manures.

Wave number (cm^−1^)	Sample time			
	Absorption degree (%)			
	0 week	3th week	6th week	9th week
**Chicken manure**				
1450	22.35	19.32	18.15	17.16
1600	19.11	17.23	15.33	13.51
3050	12.33	12.02	11.21	10.27
**Cattle manure**				
1400	12.08	11.08	10.31	9.05
1650	17.56	16.33	16.12	14.05
3000	15.41	14.33	13.28	11.36
**Pig manure**				
1380	14.71	13.56	13.15	12.22
1580	20.35	18.78	18.25	16.06
2930	19.47	16.58	15.33	14.19
**Soybean manure**				
1350	12.89	11.65	11.33	10.56
1480	15.15	14.36	13.52	11.79
2900	9.88	9.05	9.01	8.85
**Lemon peel compost**				
1320	12.21	11.55	11.08	9.98
1460	14.25	13.21	12.33	11.95
2950	12.03	11.35	10.12	9.08

**Table 7:** Types of functional groups and their strength as determined by FTIR analysis on different composts.

Composts	Strong	Medium	Weak
Chicken manure	OH group, C=C bonding, C–O or –OH(1160 cm^−1^)	COOH, C=O	CH, CH_2_, CH_3_
Cattle manure	OH group, C=C bonding, C–O or –OH(1160 cm^−1^)	COOH, C=O	CH, CH_2_, CH_3_
Pig manure	OH group, C=C bonding	COOH, C=O, C–O	CH, CH_2_, CH_3_
Soybean manure	C=C bonding	OH group, COOH, C=O, C–O	CH, CH_2_, CH_3_
Lemon peel compost	C=C bonding	OH group, COOH, C=O, C–O	CH, CH_2_, CH_3_

### ^13^C NMR analysis on five types of compost

Table 8 showed the ^13^C NMR spectroscopy of different mature composts. The five different composts were mainly composed of aliphatic group carbon and chromatic group carbon, C–O bond (CO carbons-alcohols, esters, ethers, carbohydrates), carboxyl and ester carbons and C=O bond (aldehydes and ketones) and other functional groups that were existed on the surface of composts. It provided a reference for further exploration.

**Table 8:** The ^13^C NMR spectroscopy of five different composts.

	Chicken manure (%)	Cattle manure (%)	Pig manure (%)	Soybean manure (%)	Lemon peel compost (%)
0–50 ppm	28.38	22.13	31.62	37.94	36.23
50–110 ppm	32.26	32.20	41.36	43.16	41.10
110–160 ppm^a^	16.51	10.49	17.71	18.40	17.43
160–190 ppm^b^	19.15	13.08	16.51	18.87	18.22
190-220 ppm	3.69	2.81	3.72	5.73	5.55
aliphatic C^c^	60.65	54.33	72.97	81.10	77.33

^a^Characteristic carbons in the range of chemical shift are assigned as aromatic C.

^b^Characteristic carbons in the range of chemical shift are assigned as carboxylic C.

^c^Expressed by the sum of the percentages of the characteristic carbons in the range of chemical shifts of 0–50, 50–110 ppm.

## Discussion

After composting of five composts, organic matter content was decreased from 92.5 to 64.0%, however, humic acid increased following composting time. The C/N ratio decreased were either decreased. It showed that the composts were fully mature in case of C/N ratio below 20^25^.

In general, the animal manures will decrease their pH, however, soybean manure and lemon peel compost will increase their pH after composting. It was perhaps due to basic ammonium N was decomposed by microorganism and then changed to acidic composts (e.g. chicken, cattle and pig manures)^28^. Oppositely, organic acid was gradually decomposed by microorganism in the composts of plant manures (e.g. soybean, lemon peel waste)^29^. Therefore, animal manures and plant manures showed opposite trend in pH after composting. However five compost have pH values falling in the range of slightly acid to slightly base and so soil pH will not dramatically change after application. The contents of organic matter in five composts showed gradually decreased following composting time due to organic matter was decomposed by microorganism and organic matter converts to complicated humic acid resulting in an increase of humic acid for five composts^29^. The C/N ratio decreased gradually to below 20 after 8 week composting. For this reason, FTIR and ^13^C NMR analysis were evaluated after the 8 weeks of composting time because five composts were all mature.

The absorption of five composts with FTIR analysis showed similar characteristics to previous studies^30^. According to Shin et al.^31^, the peak of aliphatic group area at 2930 cm^−1^ and 2850 cm^−1^ gradually decreased and amino acid at 1385 cm^−1^ gradually disappeared due to the decomposition of composts by microorganism. It maybe applied to determine the maturity degree of the compost. For chicken manure, the peak of absorption decreased slowly at wave number 1450 cm^−1^ following composting time. It represented amino acid content in the chicken manure was decreased due to decomposition by microorganism and getting more mature. On the other hand, the rapid decrease of amino acid in the chicken manure is probably the reason why nitrogen decrease following composting time. At wave number 1600 cm^−1^ the peak of absorption also decreased rapidly with maturity degree, it showed that the aromatic group compounds are also gradually decomposed. At were member 3050 cm^−1^, its peak of absorption slowly decreased indicating that aliphatic group compounds decomposed more slowly with maturity. For cattle manure, the wave number 1400 cm^−1^ decreased from 12.08 to 9.05% following composting time. It showed that the amino acid was slowly decomposed as maturity degree. Compared with the chicken manure, N content increased, probably due to volume compression of compost. At wave number 1650 cm^−1^ was decreased from 17.65 to 14.05% representing aromatic group compounds were gradually decomposed by microorganism. At wave number 3000 cm^−1^, the absorption slowly decreased from 15.41 to 11.36% indicating slower decomposition of aliphatic group compounds in the cattle manure when the compost was further mature. The maturity degree measurement of the cattle manure compost perhaps be set at wave number 1400 cm^−1^ (wave length about 2140 nm) with light absorption below 9%, wave number 1650 cm^−1^ (wave length about 6060 nm) with absorption below 14% and wave number 2930 cm^−1^ (wave length about 3330 nm) with absorption below 11%^32^. For pig manure, the absorption at wave number 1380 cm^−1^ decreased following composting time from 14.71 to 12.22%. The amino acid decreased due to decomposition by microorganism. The decrease was lower than that of chicken manure. The absorption decreased from 20.35 to 16.06% at wave number 1580 cm^−1^ that was similar to that of cattle manure and chicken manure. The absorption at wave number 2930 cm^−1^ decreased from 19.47 to 14.19%. Compared with chicken manure and cattle manure, the aliphatic group compounds was decomposed faster. The maturity degree measurements should be set at wave number 1380 cm^−1^ (wave length about 2200 nm) with absorption below 12%, wave number 1580 cm^−1^ (wave length 6300 nm) with absorption below 16% and wave number 2930 cm^−1^ (wave length about 3400 nm) below 14%. For soybean manure, the absorption at wave number 1350 cm^−1^ decreased from 12.89 to 10.56% following composting time, indicating amino acid was decomposed. The absorption at wave number 1480 cm^−1^ decreased from 15.15 to 11.79% indicating faster decomposition rate for aromatic group compounds than for amino acid. For lemon peel compost, the absorption at 1320 cm^−1^ decreased from 12.21 to 9.98% following composting time, indicating the decomposition of amino acid following composting time. The absorption at 1460 decreased from 14.25 to 11.95% representing decomposition of amino acid. The absorption at 2950 cm^−1^ decreased from 12.03 to 9.08% showed decomposition of aliphatic group compounds (slower than the other four composts). It is perhaps high content of fiber in the lemon peel waste, rendering slower decomposition of amino acid, aromatic group or aliphatic group.

**Figure 1:**
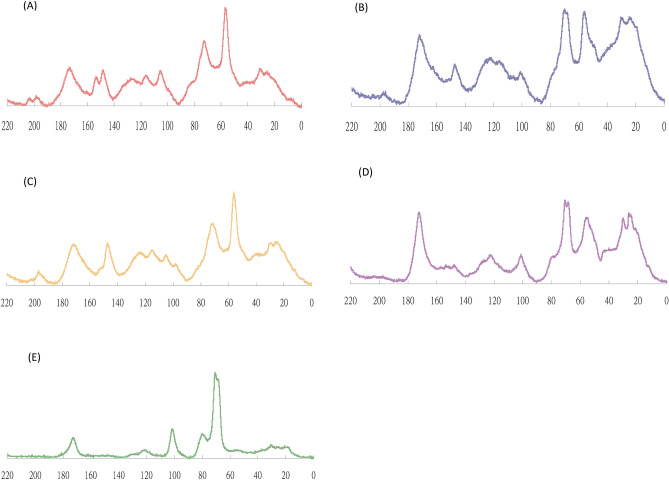
^13^C NMR graphs of five types of compost. (**A**: chicken manure, **B**: cattle manure, **C**: pig manure, **D**: soybean manure, **E**: lemon peel compost).

To understand the total functional group and their relative ratios in different materials^33^, ^13^C solid NMR spectrophotometer was applied to analyze their component and relative ratios. Figure 1 showed solid ^13^C NMR spectrophotometer analysis on different composts. It showed that the aliphatic C was higher in soybean manure and lemon peel compost, and it was lower in chicken manure, cattle manure and pig manure. Mathers and Su (2003) indicated that the four chemical shift of ^13^C NMR spectra were divided as follow^34^: alkyl C (0–50 ppm), O-alkyl C (50–110 ppm), aromatic C (110–160 ppm) and carbonyl C (160–200 ppm). In some instances, it was necessary to further divide some chemical shift regions, these were: O-alkyl C into methoxyl C (50–60 ppm), carbohydrate C (60–90 ppm) and di-O-alkyl C (90–110 ppm); and aromatic C into aryl C (110–142 ppm) and phenolic C (142–160 ppm). The chemical shift of samples of five composts were mainly distributed in 0–50 ppm, it belong to alkyl group (97.99%). The results were similar to Lin and Su (2010) for the analysis of humic acids extracted from four different composts^35^. In the experiment, it showed that five composts mainly contained fatty carbon group and aromatic carbon involved in C–O bonds of carboxyl and ester (CO Carbons-alcohols, easter, ethers, carbohydrates), On the other hand, the functional groups of C=O bonds (aldehydes and ketones). Based on FTIR and ^13^C NMR analysis, the increase and degradation of functional groups on the surface of five composts may be the clue for advanced search of appropriate wavelength to design the instrument that detect the maturity of different composts.

## Conclusion

Following composting, pH of chicken Manure, cattle Manure and pig Manure was changed from slightly base to neutral, however, soybean manure and lemon peel was changed from slightly acid to neutral. Except for chicken manure compost, nitrogen of other four composts increase gradually. By way of FTIR analysis, it showed that the nitrogen content of chicken manure was decreased due to decomposition of amino acid. After 8 weeks, five studied composts were mature because C/N ratio were getting below 20. The maturity of composts may be detected by the change of spectrum (e.g. FTIR, ^13^C NMR etc.) which the characteristics of spectrum will be used to determine the maturity of composts. It was expected to provide the clue to study further optimal conditions for the design of detecting instrument in the future.

## Data Availability

The datasets generated and/or analysed during the current study are available in the [Scientific Reports] repository, [PERSISTENT WEB LINK TO DATASETS]. The datasets used and/or analysed during the current study available from the corresponding author on reasonable request.
